# Elucidating the antiviral potential of polysaccharides

**DOI:** 10.17179/excli2022-5621

**Published:** 2023-01-06

**Authors:** Rabab Fatima, Mousmee Sharma, Parteek Prasher, Gaurav Gupta, Kumar Singh, Monica Gulati, Kamal Dua

**Affiliations:** 1 Department of Chemistry, University of Petroleum & Energy Studies, Energy Acres, Dehradun 248007, India; 2 Department of Chemistry, Uttaranchal University, Dehradun 248007, India; 3 School of Pharmacy, Suresh Gyan Vihar University, Mahal Road, Jagatpura, Jaipur, India; 4 Uttaranchal Institute of Pharmaceutical Sciences, Uttaranchal University, Dehradun, 248007, India; 5 Department of Pharmacology, Saveetha Dental College, Saveetha Institute of Medical and Technical Sciences, Saveetha University, Chennai, India; 6 School of Pharmaceutical Sciences, Lovely Professional University, Phagwara, Punjab, 144411, India; 7 Faculty of Health, Australian Research Center in Complementary and Integrative Medicine, University of Technology Sydney, Ultimo, NSW, 2007, Australia; 8 Discipline of Pharmacy, Graduate School of Health, University of Technology Sydney, Ultimo NSW, 2007, Australia

## ⁯

***“Polysaccharides present a safer and non toxic approach towards the development of new therapies and vaccines and tackling life threatening pandemics.” ***[Parteek Prasher]

The last five decades have evidenced the emergence of fatal viral outbreaks like Human Immunodeficiency Virus (HIV), Swine flu, H1N1 influenza virus, Severe Acute Respiratory Syndrome Coronavirus (SARS-CoV), Middle East Respiratory Syndrome Coronavirus (MERS-CoV), Ebola virus, Zika, Dengue, Chikungunya and most frightening Severe Acute Respiratory Syndrome Coronavirus-2 (SARS-CoV-2) (Bloom and Cadarette, 2019[2]). The treatment strategies and drugs developed to tackle the mortality rate is now least effective due to rising resistance and unprecedented mutations. Moreover, the toxicity imposed after antiviral drug intake is another challenge faced by the medical and pharmaceutical world today. There is a dire necessity for natural or plant-based antiviral medications that not only are non-toxic but also meet safe drug pharmacokinetics criteria.

Polysaccharides are natural molecules found in plants, animals, seaweeds and microorganisms. Their bioactivity depends on their composition, sugar linkages and stereochemistry (Wang et al., 2018[15] [This is a useful reference to understand the importance of sulphated polysaccharides against the pathophysiology of viral infection]). Over the decades it has been known that the presence of sulphate groups, their position and the degree of sulphation ameliorates the anti viral potential of most polysaccharides. The bioactivity of well known anti coagulant heparin sulphate, glycosaminoglycans (GAGs) and their association with S-protein of SARS-CoV-2 has been assessed on ACE2 and TMPRSS2 expressing Vero-CCL81 cells, using surface plasmon resonance. The results display a strong attachment of sulphated polysaccharides to the S protein of SARS-CoV-2 with minimal toxicity (Kwon et al., 2020[9] [This is a useful reference to study the mechanism of SARS-CoV-2 replication and further inhibition by polysaccharides]). The use of sulphated polysaccharides with varying charge densities has been reported from brown alga *S. tenerrimum* to exert a therapeutic effect on Herpes Simplex Virus (HSV). The derivative was shown to be non-toxic and safe, upon consumption (Sinha et al., 2010[12]). Interestingly, the route of administration of SARS-CoV-2 medications also holds an important role in viral inhibition. Because SARS-CoV-2 appears to attack a host of organs and tissues, the use of non-anticoagulant polysaccharides via oral or nasal route has been found to be safer as compared to remdesivir which is used intravenously (Grein et al., 2020[5]). Another study reported polysaccharide extracts from marine brown algae *Padina tetrastromatica *to be effective against HSV. The mechanism behind HSV transmission lies mainly in the quick adsorption of viral protein onto cell membranes and the interaction of heparan sulphate with viral envelope glycoproteins such as gB, gC, and gD. Interestingly sulphation methods involving (O) position in polysaccharides led to the development of various molecules exhibiting remarkable inhibition against anti-HSV *in vitro* (Karmakar et al., 2010[8]). Antiviral potential of certain algal polysaccharides like alginate has demonstrated inhibition of reverse transcriptase enzyme responsible for viral RNA replication and prevents further adsorption of viral protein to cells (Ahmadi et al., 2015[1]). *Radix Cyathulae officinalis Kuan* polysaccharide (RCPS) derived from a herbaceous perennial plant *Radix Cyathulae officinalis Kuan* demonstrated dramatic viral inhibition after phosphorylation as compared to unphosphorylated RCPS (Feng et al., 2017[3]). This highlights the importance of phosphorylation in the activity of polysaccharides.

The role of chitosan against numerous bacterial and viral infections has been elucidated previously in the literature. Chitosan is a linear polysaccharide and is derived from chitin. Various studies have demonstrated the antiviral efficacy of chitosan against human cytomegalovirus and the H1N1 influenza A virus. Anti-influenza potential of chitosan has been thoroughly investigated when chitosan was given to mice via the intranasal route. The results show that chitosan offered protection to mice given chitosan when compared to mice given normal saline (Zheng et al., 2016[17] [This is a useful reference to understand the significance of route of polysaccharide administration in influenza]). Chitosan is also effective against Coxsackie viruses, HSV-1 and Rift Valley Fever virus (RVFV) with minimum side effects and cytotoxicity. Many factors such as degree of acetylation, chemical modification, deamination, molecular weight and concentration of chitosan play an important role in proving anti-viral efficacy of chitosan. These findings along with the presence of sulphate group on chitosan structure have validated its outstanding anti-viral potential against some human viruses like Rift Valley Fever virus, HIV, SARS-CoV-2 and H1N1 ((Jaber et al., 2022[7] [This is a useful reference to understand the anti viral potential and mechanism of action of chitosan and its derivatives]). Notably N-(2- hydroxypropyl)-3-trimethylammonium chitosan chloride (HTCC), a chitosan derivative, has shown marked inhibition against SARS-CoV-2 and MERS. The mechanism revealed the association between chitosan and viral membrane protein, which further halts the entry of the viral genome into cell membranes (Milewska et al., 2021[10]). An in-depth analysis of proteins and molecules present on mucosal surfaces of cells, pre- and post-viral attack, may provide an insight into the exact mechanism of viral entry and replication. A combination study using Lactoferrin as a potent iron-binding moiety demonstrated broad-spectrum antiviral status against SARS-CoV-2, HCoV-OC43, HCoV-NL63, and HCoV-229E.The researchers hypothesized the binding of viral proteins to heparan sulphate proteoglycans HSPGs and ACE2, and confirmed the antiviral potential when used with remdesivir *in vitro* (Hu et al., 2021[6]). A thorough understanding of ADME (absorption, distribution, metabolism and excretion) profile of polysaccharides along with proteomics might prove advantageous in designing an effective and targeted drug delivery platform against dangerous zoonotic viruses.

Heparin like polysaccharides, particularly sulphated chitosan, possess the unique ability to cease the entry of the Human Papillomavirus (HPV) on the cell surface. In an attempt to investigate this behavior, 3,6 O sulphated chitosan was explored *in vitro* and has been shown to be effective against HPV by direct binding to viral capsomeres and further downregulating PI3K/Akt/mTOR pathway (Gao et al., 2018[4] [This is a useful reference to evaluate the effects of chitosan against HPV]). Another animal polysaccharide such as chondroitin sulphate derived from sea cucumber demonstrated HIV replication ceased upon entry to the cell. A common observation for inhibition of viral genetic material is the identification and successful binding of polysaccharides to the spike proteins present on the capsid of the virus. This conceptualizes the implementation of sulphate groups on polysaccharide backbone for better affinity towards viral proteins. Chondroitin sulphate apparently inhibits the spike protein and its expression in COVID 19. But sulphated polysaccharides like fucoidan and iota carrageenan derived from algae, has shown inhibitory outcome on SARS-CoV-2 protein *in vitro* (Song et al., 2020[13] [This is a useful reference to understand the role of marine polysaccharides against SARS-CoV-2]). A unique mucoadhesive chitosan based drug formulation nanotechnology offers safer delivery of COVID 19 drugs by strongly attaching to the mucosal layer of lung epithelium with sustained drug release (Prasher et al., 2022[11] [This is a useful reference to understand the significance and role of Novochizol (a polysaccharide based technology) on mucosa of pulmonary cells in viral infection]). Scallop shell powders and Korean edible calms have also been studied to predict the mechanism behind the antiviral activity of polysaccharides against avian influenza virus and HIV (Thammakarn et al., 2015[14]; Woo et al., 2001[16]). 

Polysaccharides can be future candidates for mitigating viral infections, but more *in vitro*, and *in vivo* along with promising clinical trials are needed for better outcomes. Since future viral outbreaks are quite predictable, it is important to design a non-immunogenic, non-toxic and targeted drug delivery system to effectively deliver polysaccharide based antviral drugs to the infected population without any serious side effects and quicker recovery. The benevolent nature, biocompatibility, ease of availability, low cost and non-toxic profile of polysaccharides, claim their anti-viral potential with enhanced therapeutic response. The development and discovery of polysaccharide-based drugs, antibodies and vaccines may prove beneficial in arresting episodes confronted during acute respiratory distress syndrome, particularly in COVID 19 pneumonia. Chemical modification further ameliorates the challenges faced with unmodified polysaccharides. In fact, a combination of chemically modified polysaccharides and anti viral therapeutics, with a change in route of administration may play a decisive role in combating pandemics and epidemics. In a time where viral genome sequencing has unraveled sophisticated molecular pathways responsible for the survival of viruses, genomic epidemiology along with microfluidics can also play a crucial role in mapping evolutionary behaviour and understanding the virulent nature of mutating strains. Despite significant advances in the use of polysaccharides as excipients in recent years, the exact mechanism of viral interaction and signaling pathway is not clear. This necessitates delving into high throughput screening strategies for these dangerous viral infections for tangible patient outcomes. In fact, an amalgamation across different pharma sectors along with the latest AI (artificial intelligence) tools can fully leverage the antiviral potential of polysaccharides. Identifying potent biomarkers may also help, design polysaccharide-based anti-viral drug candidates to further gain an in-depth understanding and dynamics of infection.

## Notes

Rabab Fatima, Parteek Prasher and Kamal Dua contributed equally.

## Conflict of interest

The authors declare no conflict of interest.

## References

[1] Ahmadi A, Zorofchian Moghadamtousi S, Abubakar S, Zandi K (2015). Antiviral potential of algae polysaccharides isolated from marine sources: a review. Biomed Res Int.

[2] Bloom DE, Cadarette D (2019). Infectious disease threats in the twenty-first century: strengthening the global response. Front Immunol.

[3] Feng H, Fan J, Yang S, Zhao X, Yi X (2017). Antiviral activity of phosphorylated Radix Cyathulae officinalis polysaccharide against Canine Parvovirus in vitro. Int J Biol Macromol.

[4] Gao Y, Liu W, Wang W, Zhang X, Zhao X (2018). The inhibitory effects and mechanisms of 3,6-O-sulfated chitosan against human papillomavirus infection. Carbohydr Polym.

[5] Grein J, Ohmagari N, Shin D, Diaz G, Asperges E, Castagna A (2020). Compassionate use of remdesivir for patients with severe Covid-19. N Engl J Med.

[6] Hu Y, Meng X, Zhang F, Xiang Y, Wang J (2021). The in vitro antiviral activity of lactoferrin against common human coronaviruses and SARS-CoV-2 is mediated by targeting the heparan sulfate co-receptor. Emerg Microbes Infect.

[7] Jaber N, Al-Remawi M, Al-Akayleh F, Al-Muhtaseb N, Al-Adham ISI, Collier PJ (2022). A review of the antiviral activity of Chitosan, including patented applications and its potential use against COVID-19. J Appl Microbiol.

[8] Karmakar P, Pujol CA, Damonte EB, Ghosh T, Ray B (2010). Polysaccharides from Padina tetrastromatica: Structural features, chemical modification and antiviral activity. Carbohydr Polym.

[9] Kwon PS, Oh H, Kwon SJ, Jin W, Zhang F, Fraser K (2020). Sulfated polysaccharides effectively inhibit SARS-CoV-2 in vitro. Cell Discov.

[10] Milewska A, Chi Y, Szczepanski A, Barreto-Duran E, Dabrowska A, Botwina P (2021). HTCC as a polymeric inhibitor of SARS-CoV-2 and MERS-CoV. J Virol.

[11] Prasher P, Fatima R, Sharma M (2022). Cationic polysaccharides: emerging drug delivery vehicle across the physiological mucus barrier. Future Med Chem.

[12] Sinha S, Astani A, Ghosh T, Schnitzler P, Ray B (2010). Polysaccharides from Sargassum tenerrimum: structural features, chemical modification and anti-viral activity. Phytochemistry.

[13] Song S, Peng H, Wang Q, Liu Z, Dong X, Wen C (2020). Inhibitory activities of marine sulfated polysaccharides against SARS-CoV-2. Food & Function.

[14] Thammakarn C, Tsujimura M, Satoh K, Hasegawa T, Tamura M, Kawamura A (2015). Efficacy of scallop shell powders and slaked lime for inactivating avian influenza virus under harsh conditions. Arch Virol.

[15] Wang Z, Xie J, Shen M, Nie S, Xie M (2018). Sulfated modification of polysaccharides: Synthesis, characterization and bioactivities. Trends Food Sci Technol.

[16] Woo ER, Kim WS, Kim YS (2001). Virus-cell fusion inhibitory activity for the polysaccharides from various Korean edible clams. Arch Pharm Res.

[17] Zheng M, Qu D, Wang H, Sun Z, Liu X, Chen J (2016). Intranasal administration of chitosan against influenza A (H7N9) virus infection in a mouse model. Sci Rep.

